# Farm-level livestock loss and risk factors in Ethiopian livestock production systems

**DOI:** 10.1007/s11250-025-04479-4

**Published:** 2025-06-03

**Authors:** Yin Li, Wudu Temesgen Jemberu, Dianne Mayberry

**Affiliations:** 1 CSIRO Agriculture and Food, 306 Carmody Rd, St Lucia, Australia; 2 International Livestock Research Institute, PO Box 5689, Addis Ababa, Ethiopia; 3 University of Gondar, PO Box 1191, Gondar, Ethiopia; 4 Global Burden of Animal Diseases Programme, St Lucia, Australia

**Keywords:** GBADs, Cattle, Sheep, Goats, Production loss

## Abstract

This study aims to explore farm-level losses of cattle, goats and sheep and relevant risk factors in the mixed crop-livestock and pastoral production systems in Ethiopia. Data from 1,528 cattle farms, 868 goat farms and 749 sheep farms, spanning the year 2018/19, were analysed in this study. A farm was defined as a case farm if it lost at least one cattle/goat/sheep in the past 12 months. The 12-month incidence of livestock loss was calculated for each region and production system. Logistic regression analysis was employed to assess risk factors contributing to livestock loss in the farms. Forty-five percent of goat farms, 36% of sheep farms and 23% of cattle farms reported losing at least one animal in the past 12 months. Cattle loss in the pastoral system was associated with not using vaccines (Odds Ratio = 7, *P* < 0.01). In the mixed crop-livestock system cattle loss was associated with the absence of a roofed house (Odds Ratio = 1.40, *P* < 0.05). Risk factors for goat loss in the mixed crop-livestock system were selling live goats in the past 12 months (Odds Ratio = 1.58, *P* < 0.05). For sheep loss in the pastoral system, the identified risk factor was having cattle on farm (Odds Ratio = 2.40, *P* < 0.05). These findings provide valuable insights into the scale and the drivers of livestock loss within the major cattle and small ruminants production systems in Ethiopia.

**Supplementary Information:**

The online version contains supplementary material available at 10.1007/s11250-025-04479-4.

## Introduction

Livestock industries play an important role in the Ethiopian economy, with cattle and small ruminant production contributing 14.3 billion USD (13.2% GDP) in 2021 through the provision of meat, milk, hides, and draught power (Jemberu et al. 2022; Li et al. 2023). However, despite significant investment from government and donors, high mortality rates remain a major obstacle to production, resulting in substantial financial losses for farmers and the nation (Bachewe and Tadesse 2019; Fentie et al. 2016; Tora et al. 2021a, b).

Livestock loss in Ethiopia can be attributed to various factors, including infectious diseases such as trypanosomiasis, contagious bovine pleuropneumonia, brucellosis, tuberculosis, and foot-and-mouth disease (Abdela 2017; Abdela and Yune 2017; Alemayehu et al. 2015; Shaw et al. 2014; Teklue et al. 2013; Tschopp et al. 2013). In addition to these infectious diseases, non-infectious causes like nutritional deficiencies, often arising from inadequate feeding practices, contribute to livestock loss (Kitessa et al. 2023). Beyond diseases, external stressors such as extreme weather events further increase livestock mortality (Naod et al. 2020). These factors underscore the importance of understanding the drivers behind animal health problems to implement effective livestock loss prevention and management strategies.

The causes of livestock loss in Ethiopia are influenced by the diversity of production systems, which encompass a range of breeds and practices. The dominant production system is the mixed crop-livestock system, located mainly in the highland area in the central parts of the country. Indigenous cattle are kept for draught power for crop production (Li et al. 2023), and milk from cows is largely for self-consumption. Goats and sheep are kept mainly at communal grazing within cereal crop areas or tethered in yards with feed brought to them (Jemberu et al. 2022). In the pastoral system, which is largely located in the low land area, dual-purpose indigenous cattle, sheep and goat are raised for both meat and milk.

Despite the significance of cattle and small ruminant production for the livelihoods and food security of Ethiopians, a comprehensive description of the drivers of livestock loss in different local production systems is lacking. Existing data from national surveys and databases provide a rich repository of animal health status and farm management practices at the national scale, and could offer insights on this topic. While secondary data comes with its limitations, utilising existing datasets is a cost-effective and practical approach, especially in resource-limited countries such as Ethiopia (Johnston 2014; Sorensen et al. 1996). Thus, this study seeks to investigate the incidence of livestock loss and identify management risk factors for the loss at the farm level in mixed crop-livestock and pastoral production systems using secondary data. The findings from this research will lay a foundation for understanding the key drivers behind cattle and small ruminant loss in the major livestock production systems. Furthermore, the study offers valuable insights for the attribution of disease burdens in the context of the Global Burden of Animal Diseases (GBADs) analysis (Rushton et al. 2018; Rushton et al. 2021), contributing to informed decision-making and targeted interventions in the Ethiopian livestock industry.

## Material and methods

We analysed the incidence and risk of livestock (cattle, sheep, or goat) loss at farm level in the pastoral and mixed crop-livestock zones of Ethiopia using household level data from the 2018/19 World Bank Living Standards Measurement Study (LSMS) (Central Statistical Agency of Ethiopia 2021).

The 2018/2019 LSMS survey interviewed 2,261 livestock farms, including 1,528 cattle farms, 868 goat farms and 749 sheep farms (some farms keep more than one type of livestock). The households were selected using stratified random sampling to ensure representative sampling (World Bank 2021). The LSMS data included farm-level information on livestock herd size, health, and husbandry practices such as the provision of shelter. Socioeconomic factors such as the purpose of livestock keeping, and education level of owners were also included.

The study unit for this analysis was a livestock farm. A farm that reported the loss of at least one cattle/goat/sheep in the past 12 months was defined as a case. In this instance, “loss” refers to animals leaving the herd due to death, theft, or misadventure. It does not include animals that were intentionally removed, e.g.., through sales, slaughter, or gifting. The proportion of farms that reported at least one animal lost during the 12 month period were calculated for each region (state) and production system (mixed crop-livestock or pastoral), with regions allocated to production systems based on information from the Ministry of Agriculture (Li et al. 2023). Choropleth maps were used to show the farm-level incidence risk in regions using “tmap” “sf” and “;raster” packages in R (Hijmans 2022; Pebesma and Bivand 2005; R Core Team 2021; Tennekes 2018).

Descriptive statistics were used to explore the characteristics of cattle, goat, and sheep sectors in terms of the average herd size, livestock movement, socio-economic factors and husbandry practices. Logistic regression analysis was used to identify the risk factors for livestock loss in the mixed crop-livestock and pastoral systems based on variables from the LSMS survey. The screened risk factors included farm characteristics, livestock movement, social economic factors, and husbandry practices (Table 1). Binary coding was used to categorise education level, housing type, water source and feeding source for the regression analysis. Univariable regression was used to screen the potential risk factors, and the variables with P value less than 0.2 were passed to a multivariable regression. Backward stepwise regression was used to determine which factors were included in the final model. Only the factors with a P value less than 0.05 were kept in the final model.

**Table 1:** Description of variables analysed as potential risk factors for livestock loss

Category	Variable	Type of variable	Category	Questions in LSMS survey
Farm characteristics	Herd size	Discrete	Not applicable	How many cattle/sheep/goats on farm?
	Other species on farm	Nominal	• Cattle • Sheep • Goats • Poultry	What other types of livestock species are on the farm?
Livestock movement	Introduction	Binary	Yes/No	Was there introduction of live animal from outside in the past 12 months?
	Sell livestock	Binary	Yes/No	Did you sell any live animal in the past 12 months?
Social economic factors	Purpose of having livestock	Nominal	• Sale of live animals • Sale of livestock products • Food for the family • Savings and insurance • Social status • Crop agriculture (manure, draught power)	What are the main purposes for having livestock?
	Education of head of household	Binary	• Illiterate (no school education) • Educated (Primary school education at least)	Education level of the household head
Husbandry practices	Employees on farm	Binary	Yes/No	Has this holder paid anyone outside household to help look after livestock?
	Water source	Binary	• Underground (borehole, well) • Other (rainwater, rivers, lakes)	What has been the main source of water?
	Housing type	Binary	• Roofed • No roof	In the past 12 months, what was housing system for cattle/sheep/goats used?
	Purchased water during dry season	Binary	Yes/No	During dry season, has water been paid for cattle/sheep/goats?
	Feeding source	Binary	• Mainly rely on grazing/scavenging • Mainly rely on feeding	This holder’s major feeding practices for cattle/sheep/goats
	Purchased fodder	Binary	Yes/No	In past 12 months, did you purchase any fodder for cattle/sheep/goats?
	Vaccine usage	Binary	Yes/No	In past 12 months, have you vaccinated any cattle/sheep/goats?

Confounders were checked and controlled in the final model based on author’s knowledge on local livestock industries and via checking the frequency of a factor in the “Yes” and “No” categories of another factor. For example, production system was a confounder in all the models, so the risk factors analysis was conducted by each production system. In addition, the presence of goats on farm and presence of sheep on farm were considered as confounders to each other for the cattle analysis as they are often kept together and they are very similar to cattle in terms of sharing pathogens or competing for feed, so they are combined as “presence of small ruminants”. Herd size was considered as a non-modifiable factor and is naturally associated with the likelihood of losing at least one animal, so it was kept in the final model if its P value < 0.1 as a covariate to adjust the association magnitudes of the risk factors to the interested outcome. Regions were added as a random effect in the final model. If the P value of a variable was > 0.05, the variable was removed from the final model.

The Receiver Operating Characteristic (ROC) Curves of all the logistic regression models were produced using the package “qROC” in R. The area under the ROC was calculated using the same package. The value of the area under the ROC shows the success probability of using the model to decide if a farm is a case or not when applying to the exiting dataset. It is an indicator of a model’s performance.

## Results

### Characteristics of cattle, goat, and sheep farms in Ethiopia

Average herd sizes were larger for small ruminants compared with cattle, and bigger in the pastoral system compared with the mixed crop-livestock system (Table 2). Across all species and production systems, the number of animals sold was greater than the number of animals introduced, as was the proportion of farms selling or introducing animals.

**Table 2:** Characteristics of livestock production systems in Ethiopia

	**Cattle**		**Goats**		**Sheep**	
	**Mixed**	**Pastoral**	**Mixed**	**Pastoral**	**Mixed**	**Pastoral**
***Herd numbers & composition***						
Average herd size (head) (1 st—3rd quartile)	4.78 (2–6)	8.15 (2–10)	5.47 (2–7)	19.20 (10–38)	5.08 (2–6)	9.49 (4–22)
Average number of animals introduced to herd in past 12 months (head) (1 st—3rd quartile)	0.28 (0–0)	0.13 (0–0)	2.00 (1–2)	4.76 (1–5)	2.04 (1–3)	2.60 (1–3)
Average number of animals sold in past 12 months (head) (1 st—3rd quartile)	0.47 (0–1)	0.39 (0–1)	2.52 (1–3)	5.93 (3–10)	2.63 (1–3)	3.26 (2–6)
Farms with more than one species (%)	81	87	93	78	96	99
***Socio-economic***						
Most common purpose for having livestock (% farms)	Crop agriculture (47%)	Social status (42%)	Crop agriculture (41%)	Social status (71%)	Crop agriculture (55%)	Sale of live animals (35%)
Farm owners with education (%)	17	6	15	7	17	7
***Animal husbandry***						
Farms providing roofed housing for livestock (%)	64	26	76	18	82	20
Farms vaccinating livestock (%)	48	20	24	16	26	14
Farms utilising surface water sources (dam, river, rainwater) (%)	81	68	87	79	92	77
Farms only or mostly relying on grazing and scavenging as a feed source (%)	71	87	86	95	88	95
Farms purchasing feed (%)	13	4	3	3	6	2

Reasons for keeping livestock varied between species and production systems. For all species, livestock in the mixed crop-livestock zone was primarily kept as a source of manure and draught for crop production (41–55% farms, Table 2). Much smaller numbers of farms reported family consumption (9–16%), savings and insurance (6–14%), sale of live animals (6–13%), social status (6–12%) and sale of animal products (5–9%) as the main reason for keeping livestock. While social status was the most common reason for keeping cattle and goats in the pastoral system, many farms also kept these animals for savings and insurance (25% of cattle farms, 29% of goat farms) and sale of live animals (25% of cattle farms). In comparison, sheep in the pastoral production system were commonly kept for sale of live animals (35% of farms), savings and insurance (29% of farms) and social status (25% of farms). No farms in the pastoral zone reported sale of animal products or crop agriculture as reasons for keeping ruminant livestock.

Education level of the head of the household was low across all systems (6–17%), but higher in the mixed crop-livestock zone compared to pastoral areas (Table 2).

The proportion of farms providing roofed housing (livestock kept inside the house or in a barn with a roof) was substantially greater in the mixed crop-livestock system compared with the pastoral system (Table 2). For all species and systems, most farms provided some level of housing, with only 1–11% not providing any housing (Appendix 1). Water sources were similar in both production systems, with the majority of farms relying on surface water sources (dams, rivers, rainwater harvesting) rather than underground water sources (borehole or well) (Table 2). For all species and systems, most farms relied on grazing and scavenging as the main source of feed for livestock, though the proportion of cattle farms reliant on grazing was smaller than the number of small ruminant farms reliant on grazing in both systems (Appendix 1).

Vaccination rates of livestock were low in all systems (Table 2). The highest vaccination rate was for cattle in the mixed crop livestock system (48%), with rate ranging from 14–26% for other livestock and systems.

### Distribution of loss incidence by species, production system, and region

**Fig. 1:**
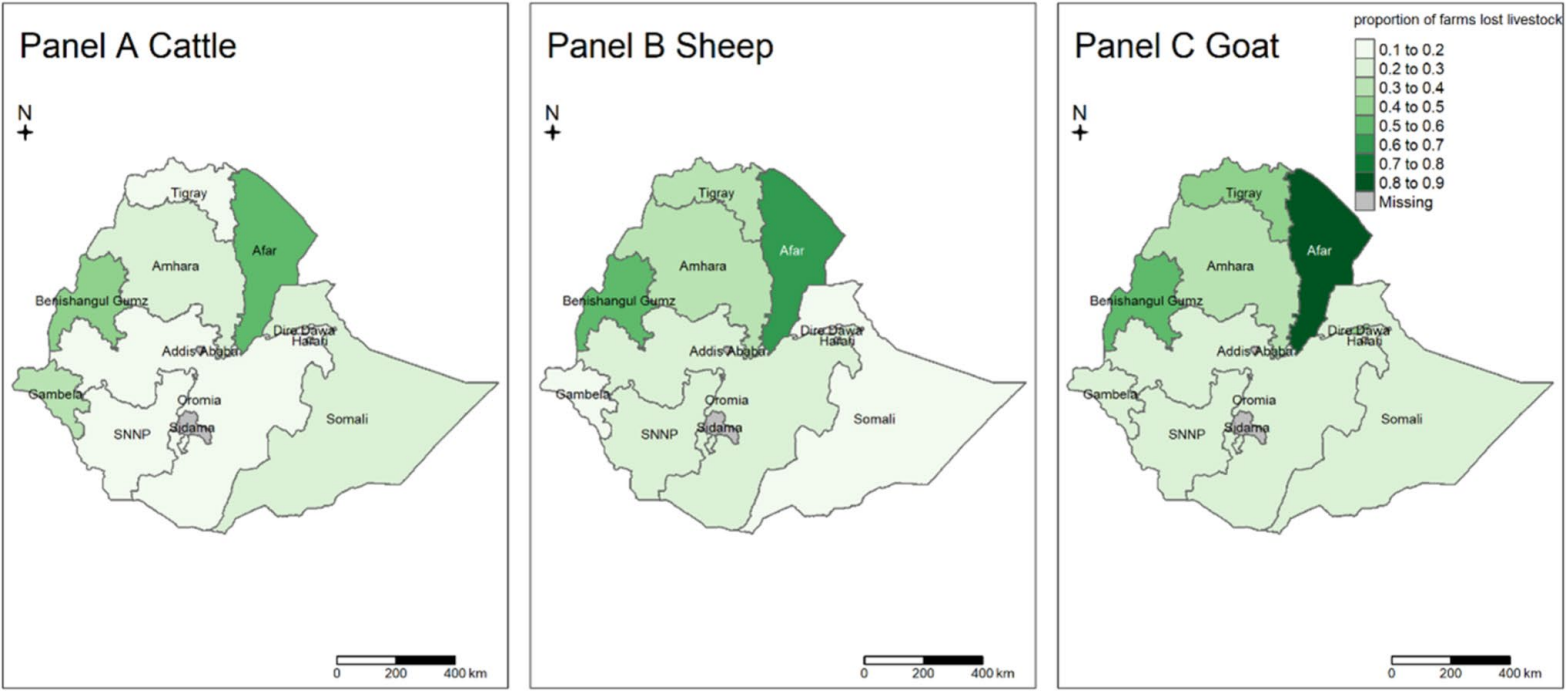
Farm-level annual Livestock loss incidence across Ethiopia. The pastoral system includes the regions of Afar, Somali, Oromia (southern part), Southern Nations Nationalities and Peoples’ Region (SNNP, southwestern part) and Gambella (western part) and the rest belong to the mixed crop-livestock system

At the national scale, the proportion of farms that reported livestock loss was highest for goat farms (45%), followed by sheep (36%) and cattle (23%) over the survey period. For all species, losses were higher in the pastoral system (goats 50%, sheep 38%, cattle 19%) compared to the mixed crop-livestock system (goats 30%, sheep 28%, cattle 19%), but there was much variation between regions within the country (Fig. 1). In general, losses tend to be higher in the north than in the south. The highest proportion of farms reporting a loss was in the Afar region for all species (goats 82%, sheep 69%, cattle 57%). The region with the lowest proportion of farms reporting a loss, for all species was Harari (sheep 0%, cattle 3%, goats 8%).

### Risk-factors for livestock loss

After characterizing the different livestock production systems, we further explored drivers for livestock loss in different systems. The risk factors for livestock loss were different between production systems and species. For cattle, provision of shelter was negatively associated with loss in the mixed crop-livestock system (Table 3), indicating that not having a roofed house for cattle is a risk factor for loss. In the pastoral system, not vaccinating cattle was a significant risk factor for cattle loss.

**Table 3:** Multivariable logistic regression model for cattle loss in the mixed crop-livestock and pastoral systems

	**Estimate**	**Std. Error**	**z value**	**Pr(>|z|)**	**Odds ratio (95% CI)**
***Mixed crop-livestock system***					
(Intercept)	−2.12	0.15	14.58	< 0.01	-
Herd size	0.17	0.02	8.51	< 0.01	1.18 (1.14–1.23)
Roofed house	−0.34	0.16	−2.06	0.04	1.40 (1.02–1.94)
Region					
*Gambela vs. Tigray*	0.85	0.44	1.93	0.05	2.35 (0.96 −5.50)
*Harar vs. Tigray*	−2.00	1.03	−1.94	0.05	0.13 (0.01 −0.66)
*Dire Dawa vs. Tigray*	0.25	0.50	0.51	0.61	1.28 (0.71 −1.95)
*Amhara vs. Tigray*	0.16	0.26	0.63	0.53	1.17 (0.71–1.95)
*Oromia vs. Tigray*	−0.15	0.26	−0.56	0.57	0.86 (0.52–1.44)
*Benishangul Gumuz vs. Tigray*	0.76	0.33	2.30	0.02	2.13 (1.11–4.07)
*SNNP vs. Tigray*	0.28	0.30	0.92	0.36	1.32 (0.73–2.38)
***Pastoral system***					
(Intercept)	−2.48	0.54	−4.53	< 0.01	-
Herd size	0.08	0.027	2.88	< 0.01	1.08 (1.03–1.14)
Not using Vaccine	1.95	0.56	3.50	< 0.01	7.00 (2.61–24.62)

For goats, herd size was a risk factor in both the mixed crop-livestock and pastoral systems (Table 4). In the mixed crop-livestock system, selling live goats in the past 12 months was risk factors. No management risk factors were significant for goat loss in the pastoral system.

**Table 4:** Multivariable logistic regression model for goat loss in the mixed crop-livestock and pastoral systems

	**Estimate**	**Std. Error**	**z value**	**Pr(>|z|)**	**Odds ratio (95% CI)**
***Mixed crop-livestock system***					
(Intercept)	0.15	0.02	6.19	< 0.01	-
Herd size	0.46	0.22	2.12	< 0.01	1.16 (1.11–1.21)
Sell live goats in the past 12 months	−0.18	0.63	−0.29	0.03	1.58 (1.03–2.42)
Region					
*Gambela vs. Tigray*	−1.17	0.54	−2.18	0.77	0.83 (0.22–2.68)
*Harar vs. Tigray*	0.44	0.42	1.05	0.03	0.31 (0.10–0.83)
*Dire Dawa vs. Tigray*	0.06	0.32	0.18	0.3	1.56 (0.67–3.58)
*Amhara vs. Tigray*	−0.25	0.34	−0.73	0.86	1.06 (0.56–2.00)
*Oromia vs. Tigray*	1.27	0.45	2.8	0.47	0.78 (0.40–1.52)
*Benishangul Gumuz vs. Tigray*	−0.38	0.37	−1.01	0.01	3.55 (1.48–8.82)
*SNNP vs. Tigray*	0.15	0.02	6.19	0.31	0.69 (0.33–1.42)
***Pastoral system***					
(Intercept)	−0.14	0.62	−0.22	0.83	-
Herd size	0.02	0.01	2.4	0.02	1.02 (1.01–1.04)
Region					
*Gambela vs. Tigray*	0.02	1.02	0.02	0.99	1.02 (0.13–7.92)
*Harar vs. Tigray*	1.03	0.64	1.61	0.11	2.80 (0.76–9.92)
*Dire Dawa vs. Tigray*	−0.36	0.89	−0.4	0.69	0.70 (0.11–3.98)
*Amhara vs. Tigray*	−1.65	0.75	−2.21	0.03	0.19 (0.04–0.82)
*Oromia vs. Tigray*	−15.47	1029.11	−0.02	0.99	*

* no CI due to highly dispersed data

For sheep, cattle raised on the farm was identified as a risk factor for loss in the pastoral system (Table 5). No other management risk factors were significant in either system.

**Table 5:** Multivariable logistic regression model for sheep loss in the mixed crop-livestock and pastoral systems

	**Estimate**	**Std. Error**	**z value**	**Pr(>|z|)**	**Odds ratio (95% CI)**
***Mixed crop-livestock system***					
(Intercept)	−1.55	0.15	−10.00	< 0.01	-
Herd size	0.12	0.02	5.54	< 0.01	1.12 (1.08—1.18)
***Pastoral system***					
(Intercept)	−1.17	0.89	−1.31	0.19	-
Herd size	0.07	0.02	3.87	< 0.01	1.07 (1.04–1.11)
Having cattle on farm	0.97	0.37	2.63	0.01	2.63 (1.29–5.48)
Region					
*Afar vs. Tigray*	0.14	0.86	0.17	0.87	1.15 (0.19–6.45)
*Oromia vs. Tigray*	−0.92	1.2	−0.77	0.44	0.40 (0.03–3.95)
*Somali vs. Tigray*	−2.09	1.01	−2.07	0.04	0.12 (0.02–0.88)
*SNNP vs. Tigray*	−1.39	1.39	−1	0.32	0.25 (0.01–3.15)

**Fig. 2:**
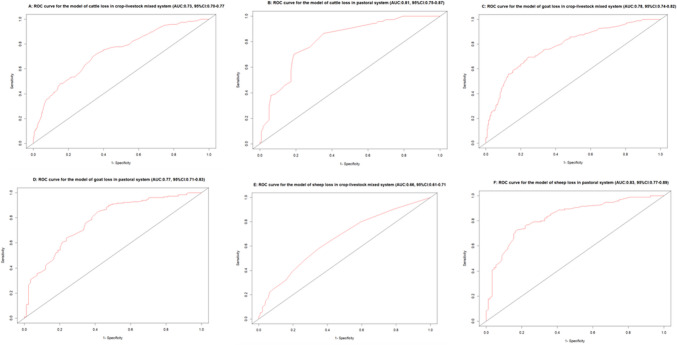
Receiver Operating Characteristic curves for multivariable regression models of cattle, goat, and sheep loss in the mixed and pastoral systems in Ethiopia

ROC analysis showed the ability of the models to discriminate between case farms and non-case farms ranged from 66 to 83% (Fig. 2). The area under the curve values for the model of loss in the mixed system and the pastoral systems were 73% and 81% (cattle), 79% and 77% (goat), 66% and 83% (sheep), respectively. The model for the sheep loss in pastoral system has the best ability (83% correctly identified) to discriminate between the case farms and the non-case farms, while the model for the sheep loss in the mixed crop-livestock system has the worst ability (only 66% correctly identified) to do so.

## Discussion and conclusion

This study highlights a high incidence of livestock loss and a diversity of management risk factors for ruminant livestock loss in Ethiopia. While some risk factors like herd size were common across multiple systems and species, the majority of risk factors were species or system specific. Although a previous study has reported risk factors for calf and lamb mortality, the study focused animal-level risk factors such as provision of colostrum to neonates, and was conducted only in one state (Hadgu et al. 2021) A national analysis on risk factors at household level was lacking. This analysis therefore offers new insights into the reasons for loss of cattle and small ruminants in different production systems.

The distribution of cattle and small ruminants loss is heterogeneous between regions in Ethiopia, indicating potential differences in disease prevalence and the presence of other hazards. The highest loss incidence was in Afar for all three species, indicating poor livestock health status or poor livestock management in this region. Afar is dominated by pastoralists, who are vulnerable to drought and disease outbreaks. In a study of pastoralists in the Afar region, Jones et al. (2020) reported that disease occurrence was associated with malnutrition and adverse weather in local livestock farms, and disease increased during the dry season. More broadly, the higher incidence of cattle and small ruminants loss observed in the pastoral system in our analysis could be due to extreme climate conditions in the low land arid and semi-arid areas of Ethiopia. For example, the annual precipitation in lowland area is less than 300 mm, compared to over 2000 mm in the highland area (World Bank 2023). In addition, feed sources are more diverse in the mixed system than in the pastoral system, as crop farmers can use crop residues as feed in dry season (Birch 2018). The remoteness of pastoral systems and limited access to animal healthcare services may also contribute to greater losses.

Drivers of loss also vary between species and production systems, highlighting the need for differing and targeted interventions. For example, not vaccinating cattle was only a significant risk factor for cattle in the pastoral system (Table 3), despite similarly low levels of vaccination for small ruminants (Table 2). This could indicate that there are differences in the specific diseases or broader causes of mortality impacting small versus large ruminants, and therefore the relevance of vaccination campaigns. Similarly, having multiple species on farm was common in all systems (Table 2), yet “having cattle on farm” was a significant risk factor only for sheep in the pastoral system (Table 4). This might reflect the different consumption habits of sheep (browsing versus grazing), a susceptibility to parasites associated with grazing, or that they are more prone to theft, predation, or misadventure. For example, diseases such as foot-and-mouth disease and Brucellosis can be shared between cattle and small ruminants (Legesse et al. 2013; Musallam et al. 2015).

The impacts of livestock loss are potentially enormous given the importance of cattle and small ruminants to Ethiopian farmers. The findings in this study would contribute to overcoming this problem by supporting disease burden attributions to different causes, namely infectious diseases, non-infectious diseases, and external factors (Larkins et al. 2023; Rushton et al. 2021). Through the GBADs program, this information can then be used by stakeholders such as the Ethiopian Government, to inform investments into the prevention and management of animal health problems. The diverse factors associated with livestock loss identified in this analysis highlight the need for the GBADs analysis to be conducted at sub-national levels.

The finding of this study may also benefit improvement of livestock production in other African countries with similar production systems. For example, the pastoral farming systems in Ethiopia are also seen in Mali, Niger, Chad, Sudan, Kenya and Uganda, and similar highland mixed systems also exit in Eritrea, Lesotho, Angola, Cameroon and Nigeria (Dixon and Gulliver 2001). It would be meaningful to compare reasons for livestock loss in the same production system between Ethiopia and its neighbouring countries. However, this was not possible in this analysis due to inconsistencies in the LSMS survey questionnaires used in different countries (Central Statistical Agency of Ethiopia 2021; World Bank 2001).

While this study provides an initial assessment of risk factors for livestock loss in Ethiopia, the results are limited by the scope of the data available. The reporting of livestock losses in the LSMS dataset did not distinguish between different types of loss (e.g., mortality or theft), or provide any detail on the causes of losses (e.g., illness, predation, malnourishment). This information is essential for a more detailed assessment, but such data rarely exists in large-scale or publicly accessible databases. The LSMS survey results may also be affected by recall bias as the farmers were asked events occurring in the past year.

## Supplementary Information

Below is the link to the electronic supplementary material.

Supplementary File 1 (docx.)

## Data Availability

The datasets generated during and/or analysed during the current study are available in the world bank repository: https://www.worldbank.org/en/programs/lsms.
