# Supplementary material for: Farm-level livestock loss and risk factors in Ethiopian livestock production systems
Source: Trop Anim Health Prod. 2025 Jun 3;57(5):240. doi: 10.1007/s11250-025-04479-4 (PMC12134002; doi:10.1007/s11250-025-04479-4)
Supplement: Supplementary file 1 — (docx.) [file 11250_2025_4479_MOESM1_ESM.docx]

Appendix 1

Table 1 Housing types of livestock farms in the two production systems

| Species | Production system | Housing type | | | | | P value |
| --- | --- | --- | --- | --- | --- | --- | --- |
|  |  | Inside the house (e.g.  Kitchen) | In barn with roof | In barn without roof | Other | None |  |
| Cattle | Mixed crop-livestock system | 37% | 27% | 24% | 1% | 11% | <0.01 |
|  | Pastoral system | 9% | 17% | 65% | 1% | 8% |  |
| Goat | Mixed crop-livestock system | 47% | 29% | 17% | 1% | 6% | <0.01 |
|  | Pastoral system | 2% | 16% | 79% | 0% | 3% |  |
| Sheep | Mixed crop-livestock system | 51% | 31% | 11% | 1% | 6% | <0.01 |
|  | Pastoral system | 4% | 16% | 79% | 0% | 1% |  |

Table 2 Water sources of livestock farms in the two production systems

| Species | Production system | Water sources | | | | | | P value |
| --- | --- | --- | --- | --- | --- | --- | --- | --- |
|  |  | Borehole | Dam | Well | River/spring/stream | Rainwater/harvesting | Others |  |
| Cattle | Mixed crop-livestock system | 11% | 4% | 6% | 75% | 2% | 3% | <0.01 |
|  | Pastoral system | 20% | 1% | 11% | 62% | 5% | 1% |  |
| Goat | Mixed crop-livestock system | 15% | 3% | 7% | 66% | 3% | 6% | <0.01 |
|  | Pastoral system | 25% | 0% | 10% | 60% | 4% | 1% |  |
| Sheep | Mixed crop-livestock system | 8% | 3% | 5% | 75% | 2% | 6% | <0.01 |
|  | Pastoral system | 24% | 1% | 9% | 60% | 5% | 0% |  |

Table 3 Feeding sources of livestock farms in the two production systems

| Species | Production system | Feeding source |  |  |  |  | P value |
| --- | --- | --- | --- | --- | --- | --- | --- |
|  |  | Only grazing/scavenging | Mainly grazing/scavenging with some feeding | Mainly feeding with some grazing/scavenging feeding with some | Only feeding (zero grazing/scavenging) | Other |  |
| Cattle | Mixed crop-livestock system | 26% | 45% | 23% | 3% | 3% | <0.01 |
|  | Pastoral system | 63% | 24% | 7% | 4% | 2% |  |
| Goat | Mixed crop-livestock system | 51% | 35% | 8% | 2% | 4% | <0.01 |
|  | Pastoral system | 87% | 8% | 3% | 1% | 1% |  |
| Sheep | Mixed crop-livestock system | 52% | 36% | 8% | 1% | 3% | <0.01 |
|  | Pastoral system | 87% | 8% | 2% | 2% | 0% |  |
